# The addition of abemaciclib to sunitinib induces regression of renal cell carcinoma xenograft tumors

**DOI:** 10.18632/oncotarget.19618

**Published:** 2017-07-27

**Authors:** Jeffrey Small, Erik Washburn, Karmaine Millington, Junjia Zhu, Sheldon L. Holder

**Affiliations:** ^1^ Division of Hematology/Oncology, Penn State Hershey Cancer Institute, Hershey, PA, USA; ^2^ Department of Pathology and Laboratory Medicine, Penn State Hershey Medical Center, Hershey, PA, USA; ^3^ Department of Public Health Sciences, Penn State University College of Medicine, Hershey, PA, USA

**Keywords:** PIM1 kinase, CDK4/6 kinase, abemaciclib, renal cell carcinoma, sunitinib

## Abstract

Multiple therapies currently exist for renal cell carcinoma, however, most do not result in cure and the development of acquired resistance is the rule rather than the exception. CDK4/6 and PIM1 kinases are potential new therapeutic targets in RCC. Abemaciclib is a potent CDK4/6 and PIM1 kinase inhibitor, thus we evaluated the effects of abemaciclib on renal cell carcinoma. *In vitro*, abemaciclib causes decreased cellular viability, increased apoptosis, and alterations in autophagy in renal cell carcinoma cell lines. A pre-clinical mouse model of RCC shows abemaciclib in combination with sunitinib to cause dramatic reduction in tumor sizes without overt toxicity. Thus abemaciclib is active in renal cell carcinoma and should be evaluated in a clinical trial in combination with sunitinib. Additionally, CDK4/6 and PIM1 kinase appear to be viable clinical targets in renal cell carcinoma.

## INTRODUCTION

Kidney cancer will account for approximately 63,990 newly diagnosed cases of cancer in the USA in 2016 [1]. The significant majority of these cases are renal cell carcinoma (RCC) with clear cell histology. Up to 30% of patients will have metastases at the time of diagnosis and approximately 50% of patients who undergo nephrectomy (radical or partial) will experience disease recurrence [2]. Current therapy for metastatic RCC (mRCC) falls into two broad categories – immunotherapy and targeted therapy. Immunotherapy with high dose interleukin-2 (IL-2) can result in durable complete responses [3–5], however the therapy it is very toxic and many if not most patients are ineligible for IL-2 therapy due to co-morbidities. Additionally, only 5-10% of patients achieve a durable complete response [3–5]. Immunotherapies available for RCC have been reviewed and new more tolerable immunotherapies are currently being investigated [6]. One such therapy, nivolumab, has shown clinical benefit [7] and is now approved for clinical use in the second line setting and beyond. Combination immunotherapy is also showing promise. In particular the combination of nivolumab and ipilimumab has shown promise in early studies in first line, second line, and beyond. However, the improved responses over nivolumab therapy are also associated with increased toxicities. A large phase III trial of this combination therapy is currently recruiting (CheckMate 214; NCT02231749).

There are numerous targeted therapy agents approved for clinical use in mRCC. These agents target the vascular epithelial growth factor (VEGF) pathway (eg., sunitinib, pazopanib, axitinib, cabozantinib, lenvatinib, sorafenib, and bevacizumab) or are mammalian target of rapamycin (mTOR) inhibitors (eg., temsirolimus, everolimus). Current systemic therapies for renal cell carcinoma have recently been reviewed [8]. Sunitinib is approved by the United States Food and Drug Administration (FDA) for first line therapy in mRCC [9]. Sunitinib targets multiple kinases including the VEGF receptor kinase, platelet-derived growth factor (PDGF) receptor kinase, cKIT, and FLT3 [10]. Sunitinib is itself active and also is metabolized to an active metabolite, SU12662, and excreted mostly in the feces [11, 12].

Most patients receiving targeted therapy develop acquired resistance and experience subsequent tumor progression. Additionally, a subset of patients experience primary resistance, having no response to initial therapy. Thus the clinical question for patients receiving these agents is not will their tumor progress, but how long until their tumor progresses. Unfortunately disease progression is followed by eventual clinical decline and ultimately death. Consequently, additional therapies are sorely needed for mRCC. In particular, new clinically relevant targets that are vulnerable to pharmacologic intervention are necessary to improve disease control and potential cure in this patient population.

Proviral Integration site of Moloney murine leukemia virus 1 (PIM1) kinase is a serine/threonine kinase that promotes cell cycle progression and inhibits apoptosis [13]. SGI1776 is a selective inhibitor of PIM1 kinase (IC_50_ 7 nM). It also has activity against PIM2 (IC_50_ 363 nM), PIM3 (IC_50_ 69 nM), Flt-3 (IC_50_ 44 nM), haspin (IC_50_ 34 nM), c-Kit, and TrkA [14]. Use of SGI-1776 has been shown to be effective against RCC cell lines and in a pre-clinical mouse model. Mice with xenograft RCC tumors were treated with vehicle, sunitinib, SGI-1776, or the combination of sunitinib and SGI-1776. After three weeks of therapy tumors were statistically significantly smaller in the sunitinib and SGI-1776 (monotherapy) cohorts than in the vehicle cohort. Moreover, tumors in the sunitninb + SGI-1776 (combination) cohort were statistically significantly smaller than tumors in either monotherapy cohort [15]. These data suggest that PIM1 kinase is a therapeutic target in RCC and that inhibition of PIM1 kinase may improve the activity of sunitinib therapy in RCC.

The CDK4/6 kinases have also been identified as potential targets in RCC. Loss of von Hippel – Lindau (VHL) protein function is a common and well described mechanism associated with the development of RCC [16]. Loss of VHL has been shown to result in up-regulation of cyclin dependent protein kinase 6 (CDK6) [17] and cyclin D1, the binding partner for CDK4/6 [17, 18]. Additionally, a selective CDK4/6 inhibitor has been shown to cause decreased proliferation of RCC cell lines [19]. Thus the CDK4/6 kinases are potential therapeutic targets in RCC. To our knowledge, there has been no prior investigation of dual targeting of CDK4/6 kinase and the VEGF pathway in RCC.

Small molecules, such as flavopiridol, that exhibit dual inhibitory activity against cyclin-dependent kinases and PIM1 kinase have been reported previously [20]. Abemaciclib is a selective CDK4/6 kinase inhibitor, with an IC_50_ of 2 nM for CDK4 and 10 nM for CDK6 [21]. Interestingly, abemaciclib is also a potent PIM1 kinase inhibitor with an IC_50_ of 50 nM [21] and much less potent PIM2 kinase inhibitor with an IC_50_ of 3400 nM [21]. Activity against PIM3 has not been reported.

Abemaciclib has been shown to be safe and tolerable in human studies in breast, lung, and additional solid tumor types [22]. Multiple phase III studies are in progress to assess efficacy: MONARCH 2 (breast cancer; NCT02107703), MONARCH 3 (breast cancer; NCT02246621), NCT02763566 (breast cancer), and JUNIPER (non-small cell lung cancer; NCT02152631). Notably, abemaciclib has received breakthrough therapy designation status from the FDA for treatment of advanced breast cancer.

With the knowledge that PIM1 and CDK4/6 kinases may be effective targets in RCC, and that PIM1 kinase inhibition improves the activity of sunitinib in RCC, we hypothesized that abemaciclib (a dual PIM1-CDK4/6 inhibitor) will be an active agent in RCC and that it may improve the activity of sunitinib when used in combination. Given the current data on abemaciclib use in other tumor types we also anticipated that positive results will have excellent translatability to the clinic. Here we report the effects of abemaciclib on RCC *in vitro* and in a pre-clinical mouse model of RCC.

## RESULTS

### PIM1 kinase is active in human RCC

**Figure 1:**
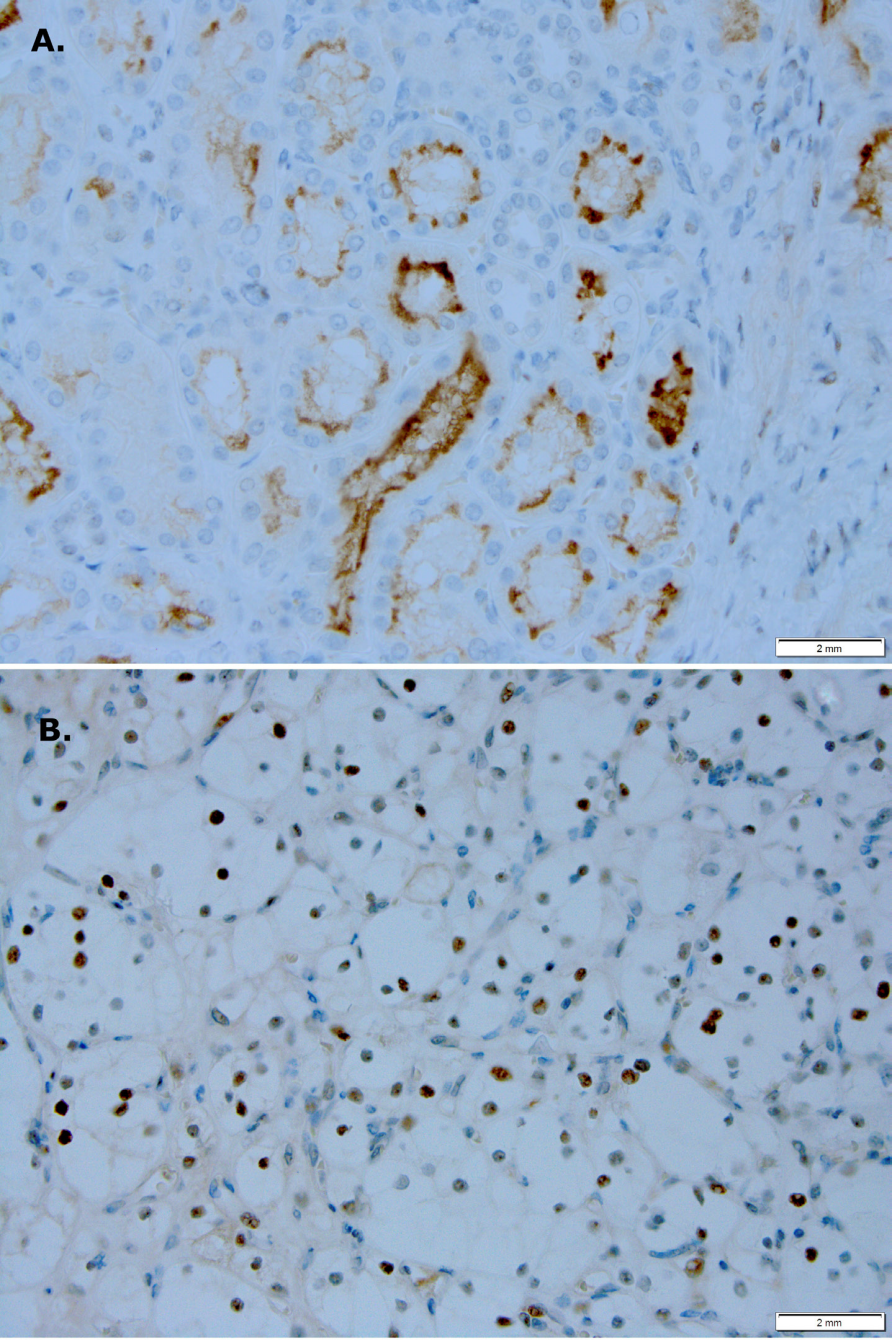
PIM1 kinase expression is different in RCC versus normal renal tissue **(A)** Focal apical membrane staining in renal tubules is seen in normal renal tissue (magnification 400x). **(B)** Focal nuclear staining is seen in RCC (magnification 400x).

To evaluate for a difference in PIM1 kinase activity in human RCC, we performed immunohistochemistry on RCC and normal adjacent tissue (NAT) obtained from archived patient nephrectomy specimens. As shown in Figure 1A, there is focal apical membrane staining of PIM1 in renal tubules in normal tissue. Four of five cases evaluated showed a similar staining pattern. In contrast, Figure 1B shows focal nuclear staining of PIM1 in RCC tissue. Focal nuclear staining was observed in four of five cases evaluated.

To further explore this idea we obtained a tissue microarray (TMA) consisting of 90 cases of RCC with 90 matched NAT specimens. Staining of the TMA showed 26% of RCC had high PIM1 staining (grade 3 or 4), while only 1% of NAT showed grade 3 and no NAT showed grade 4 staining for PIM1 (Table 1). These data suggest an oncogenic/oncosupportive process involving PIM1 in a subset of RCC cases.

**Table 1:** PIM1 kinase levels are increased in a subset of RCC

	Grade 0	Grade 1	Grade 2	Grade 3	Grade 4
RCC	37 (41%)	18 (20%)	11 (12%)	12 (13)	12 (13)
NAT	13 (14%)	63 (70%)	13 (14%)	1 (1%)	0 (0%)

RCC = renal cell carcinoma. NAT = normal adjacent tissue. Grades reflect level of PIM1 kinase staining by immunohistochemistry.

**Figure 2:**
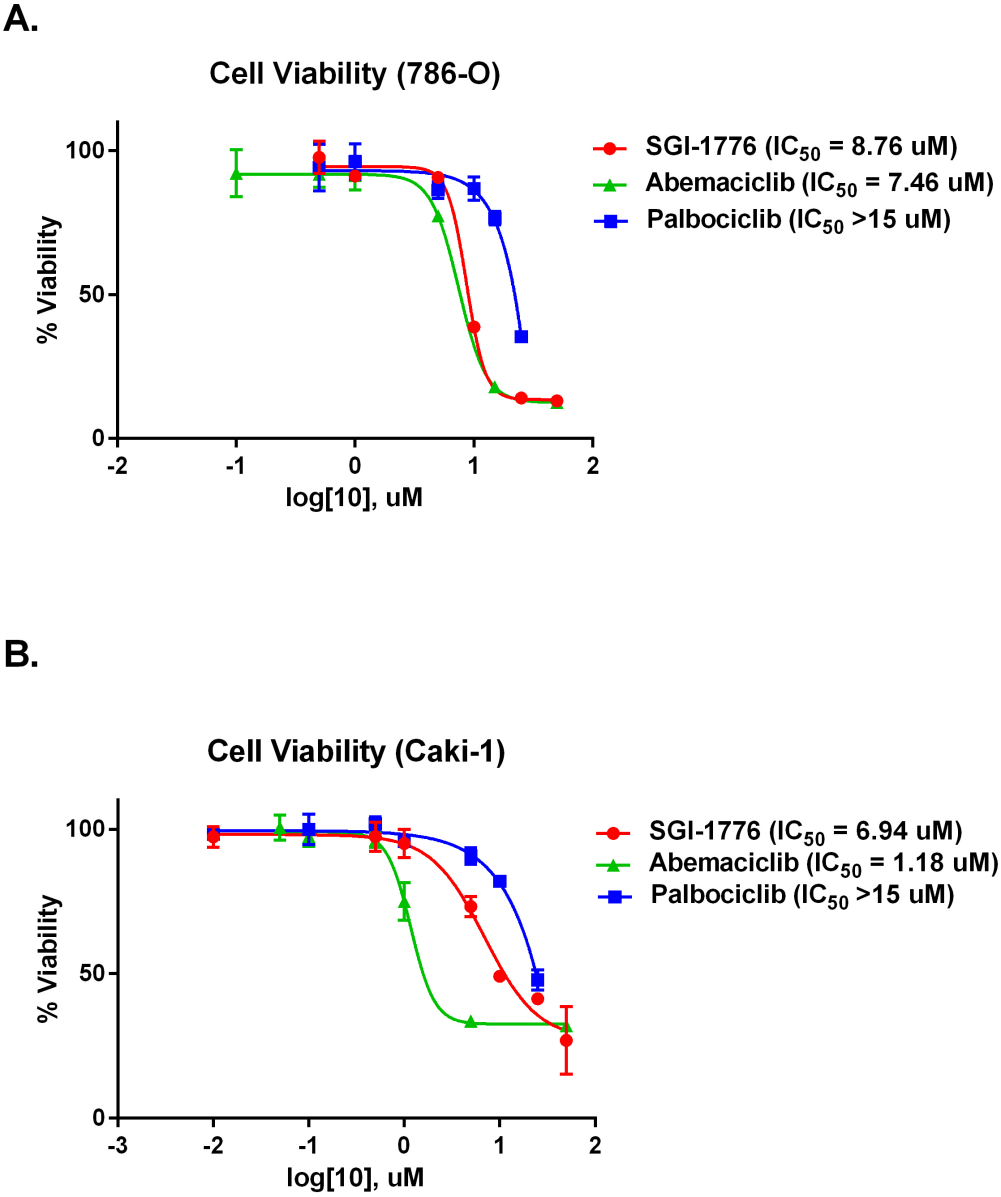
Abemaciclib causes decreased cellular viability in RCC cell lines RCC cell lines were exposed to increasing concentrations of SGI-1776, abemaciclib, or palbociclib and cell viability determined by MTT assay. **(A)** 786-0 cells. **(B)** Caki-1 cells.

To evaluate the effectiveness of PIM1 kinase as a target in RCC we performed cell viability assays on RCC cell lines. 786-O and Caki-1 cells have both been shown to have increased protein levels of PIM1 kinase compared to normal renal proximal tubule cells [15]. We determined the effect of PIM1 inhibition in each cell line using the PIM1 inhibitor SGI-1776 and compared this effect to treatment with abemaciclib (CDK4/6 and PIM1 inhibitor). Palbociclib is a selective CDK4/6 (IC_50_ 11 nM and 16 nM, respectively) kinase inhibitor with little to no activity against a panel of 36 additional kinases [23]. Palbociclib was used as a control for the effect of CDK4/6 inhibition on cell viability. Palbociclib has little direct PIM1 kinase inhibitory activity, with an IC_50_ of >10 μM for PIM1 kinase [24]. 786-O cells are VHL deficient while Caki-1 cells are VHL intact. Figure 2A demonstrates that in 786-O cells, the effect of abemaciclib (IC_50_ 7.46 μM) on cell viability is similar to SGI-1776 (IC_50_ 8.76 μM), with palbociclib (IC_50_ > 15 μM) having effects on cell viability but only at the highest concentrations tested. Caki-1 cells are most sensitive to abemaciclib exposure (IC_50_ 1.18 μM), followed by SGI-1776 (IC_50_ 6.94 μM) and are sensitive to palbociclib (IC_50_ > 15 μM) at the highest concentrations tested (Figure 2B).

### Combining PIM1 inhibition with sunitinib is superior to monotherapy

**Figure 3:**
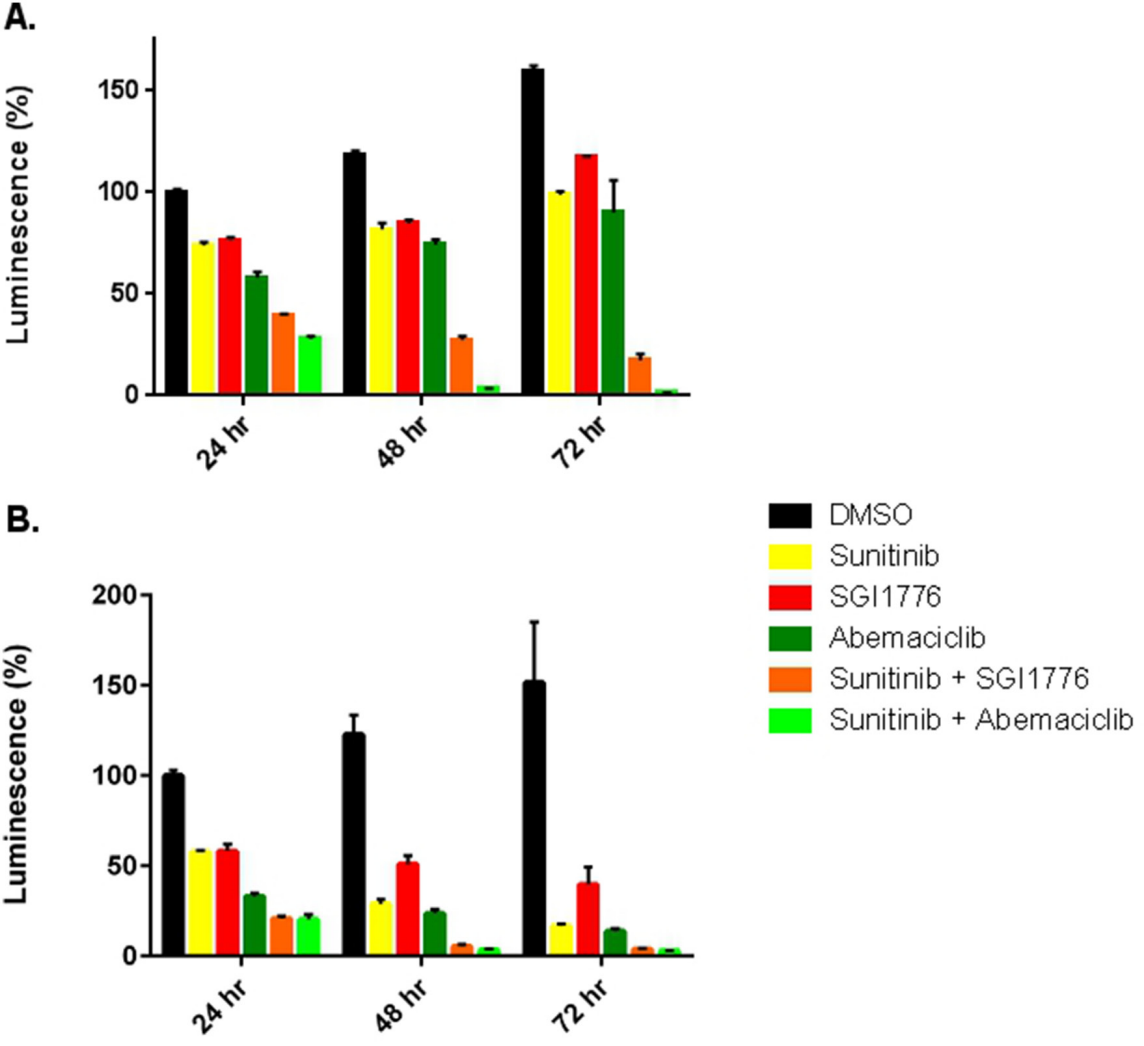
Abemaciclib is more effective against RCC cell lines in combination with sunitinib 786-O **(A)** and Caki-1 **(B)** cells were treated with single agent therapy, or in combination with sunitinib. Cellular viability was determined by CellTiter-Glo^®^.

Prior reports have shown increased efficacy of sunitinib in RCC when combined with a PIM1 kinase inhibitor [15]. Thus we evaluated cell viability in 786-O and Caki-1 cells when exposed to sunitinib, abemaciclib, or both drugs in combination. 786-O cells treated with abemaciclib showed decreased viability at 24, 48, and 72 hours compared to vehicle (Figure 3A). Similar effects on viability were observed with sunitinib and SGI-1776. Both abemaciclib and SGI-1776, when used in combination with sunitinib, result in a more rapid and more effective cellular effect than either drug as monotherapy. Similar results were observed in Caki-1 cells (Figure 3B). Pair-wise comparisons of cell viability between drug therapies were statistically significant the vast majority of the time, except for in Caki-1 cells at the 72 hour time point where all therapies were significantly different from DMSO but not from each other (Supplementary Table 1).

We also performed a formal evaluation for synergy using the Chou-Talalay method [25]. The combination index for the combination abemaciclib/sunitinib suggests a synergistic effect at the low concentrations used in our *in vitro* studies (Supplementary Table 2). At higher concentrations the effect appears to be additive.

We also determined the effect of increasing concentrations of abemaciclib, SGI-1776, or palbociclib, in combination with a constant concentration of sunitinib. As expected, cellular viability decreased with increasing concentrations of abemaciclib or SGI-1776. Effects of palbociclib were only seen at the highest concentrations tested. Results were similar in 786-O and Caki-1 cells. (See Supplementary Figures 1 and 2).

### Combination abemaciclib/sunitinib increases apoptosis and induces changes in autophagy

**Figure 4:**
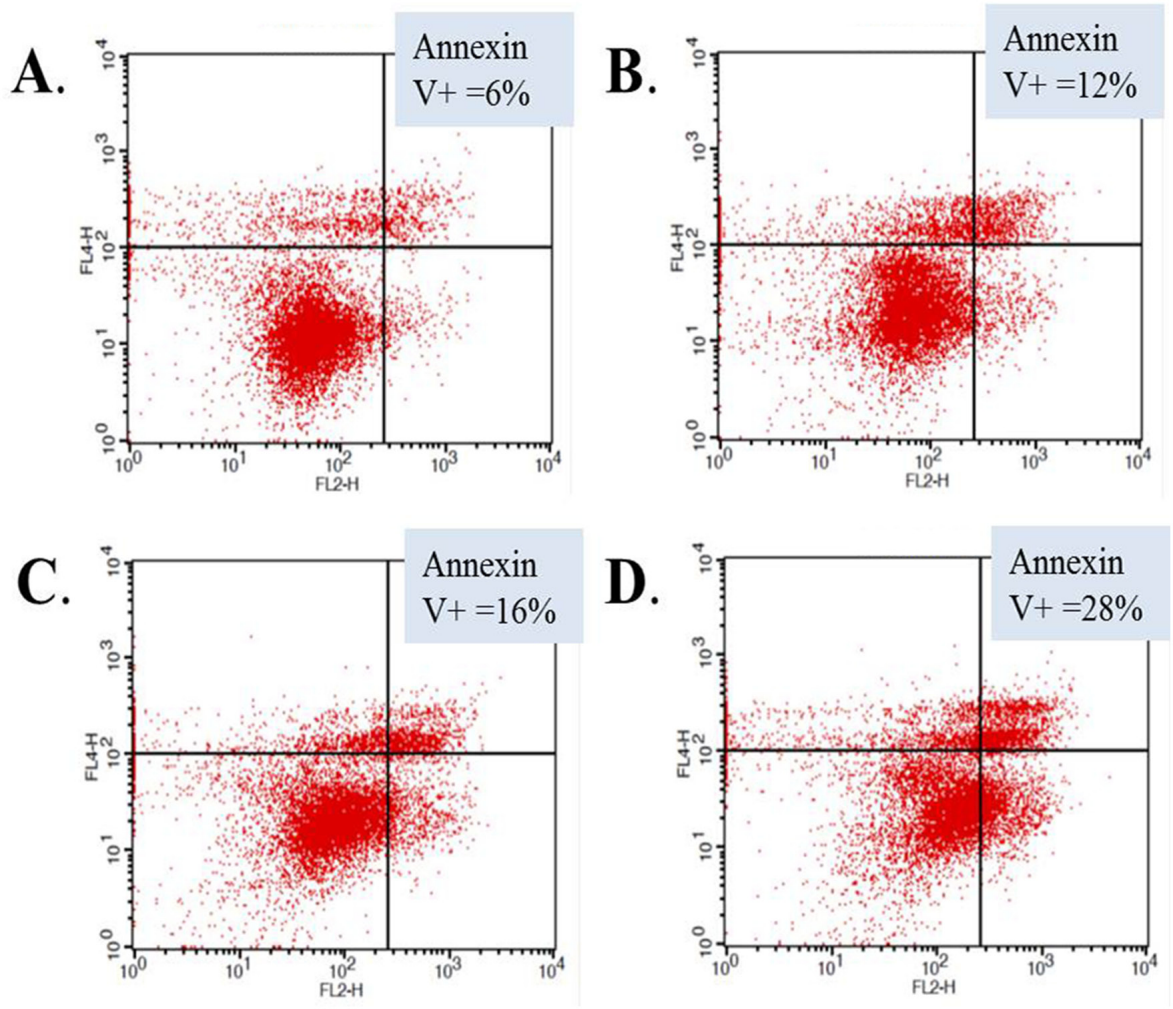
Abemaciclib induces increased apoptosis in RCC cells 786-O cells were treated with DMSO **(A)**, sunitinib **(B)**, abemaciclib **(C)**, or abemaciclib + sunitinib **(D)**. Cells were stained for annexin V and positivity determined by flow cytometry.

We performed additional experiments to elucidate possible mechanisms of the observed cellular effects of abemaciclib on RCC cell lines. We treated 786-O cells with sunitinib, abemaciclib, or the combination and evaluated changes in annexin V staining to determine the effects of each drug and the combination on apoptosis. Figure 4 shows annexin V staining was increased in cells treated with sunitinib and in cells treated with abemaciclib, suggesting an increase in apoptosis as a result of exposure to each drug alone. When cells were treated with abemaciclib and sunitinib in combination, annexin V staining was greater than with either drug alone. These data suggest an increase in apoptosis as a possible mechanism for the cellular effects of abemaciclib, and combination abemaciclib/sunitinib on 786-O cells.

**Figure 5:**
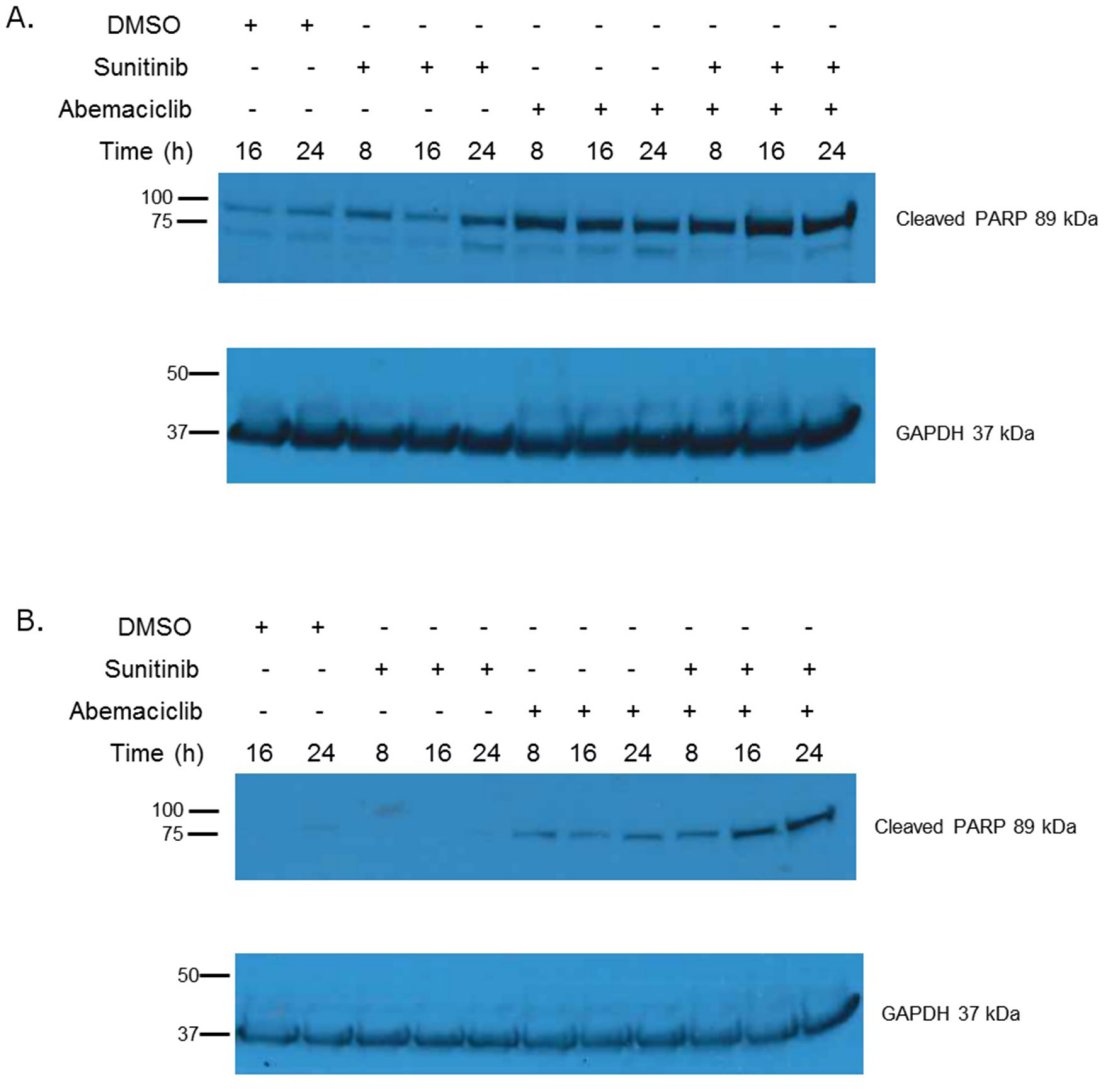
Abemaciclib causes increased PARP cleavage in RCC In 786-O cells **(A)** and Caki-1 cells **(B)** abemaciclib exposure results in increased PARP cleavage. This effect is more rapid and pronounced when abemaciclib is combined with sunitinib.

We also evaluated cleavage of poly ADP-ribose polymerase (PARP) as an additional means of determining changes in apoptosis. Immunoblot assays show that PARP cleavage is increased in a time-dependent manner when RCC cell lines are exposed to abemaciclib (Figure 5). Interestingly, PARP cleavage is more rapid and pronounced when abemaciclib is combined with sunitinib. These data further suggest that abemaciclib causes increased apoptosis in RCC cell lines, with this effect being amplified by combination with sunitinib.

Due to its CDK4/6 inhibitory activity, abemaciclib may also affect cell cycle progression. Thus we used flow cytometric analyses to determine the effect of abemaciclib on 786-O cells. Abemaciclib caused an increase in the population of cells in S-phase of the cell cycle (Supplementary Figure 3C) but did not appear to cause G1 arrest. The combination of abemaciclib and sunitinib did not appear to alter the effects of abemaciclib on cell cycle progression in 786-O cells (Supplementary Figure 3D).

**Figure 6:**
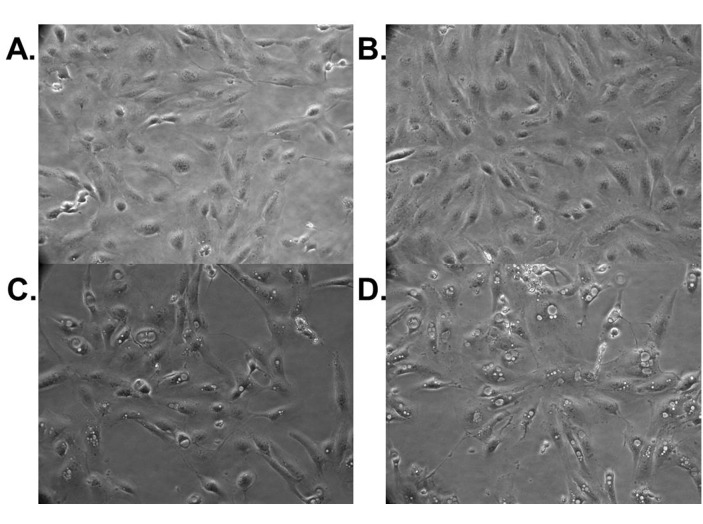
Abemaciclib causes morphologic changes in RCC cell lines 786-O cells were treated with DMSO **(A)**, sunitinib **(B)**, abemaciclib **(C)**, or abemaciclib + sunitinib **(D)**. Pictures were taken after 24 hours of treatment. Magnification factor is 20X.

During our experiments we noted morphologic changes in RCC cell lines induced by treatment with abemaciclib. Figure 6 shows the development and accumulation of vacuoles in 786-O cells treated with abemaciclib for 24 hours. Vacuolization is more prominent when cells are treated with abemaciclib in combination with sunitinib, while very little vacuolization is seen in cells treated with sunitinib alone. Similar results were observed in Caki-1 cells (data not shown).

**Figure 7:**
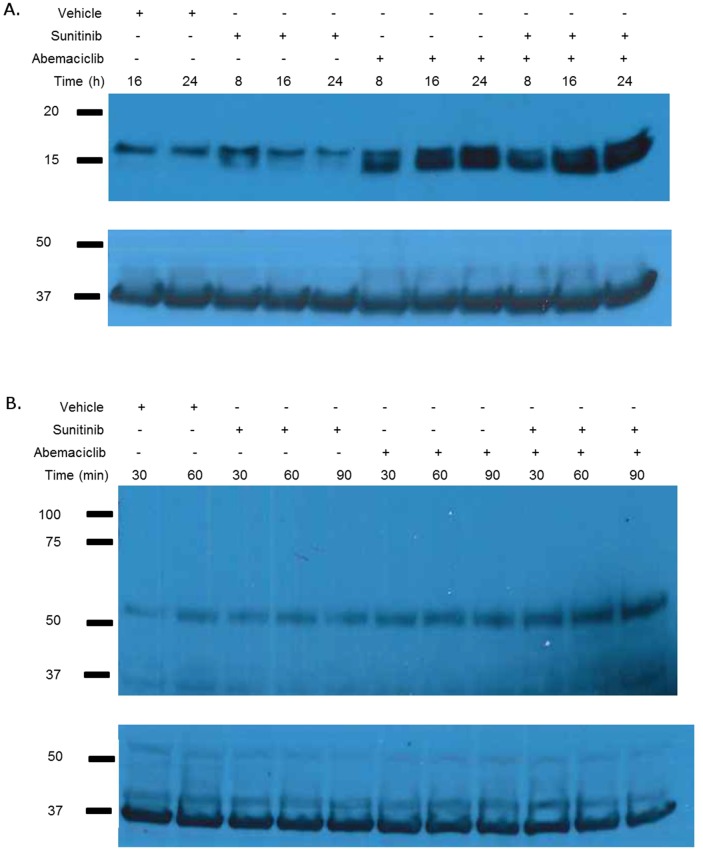
Abemaciclib induces changes in markers of autophagy 786-O cells were treated as labelled and immunoblot analysis performed on whole cell lysate for LC3a/b **(A)** and beclin-1 **(B)**. Abemaciclib causes a time-dependent increase LC3b and beclin-1.

The observed morphologic changes are not consistent with the process of apoptosis; however, they are reminiscent of autophagy. Consequently we evaluated the effects of abemaciclib on markers of autophagy. Figure 7A shows abemaciclib causes a time-dependent increase in LC3B. We also observed an abemaciclib induced time-dependent increase in beclin (Figure 7B). These data suggest that abemaciclib induces changes in autophagy.

### Combination abemaciclib/sunitinib therapy causes rapid tumor regression in mice

**Figure 8:**
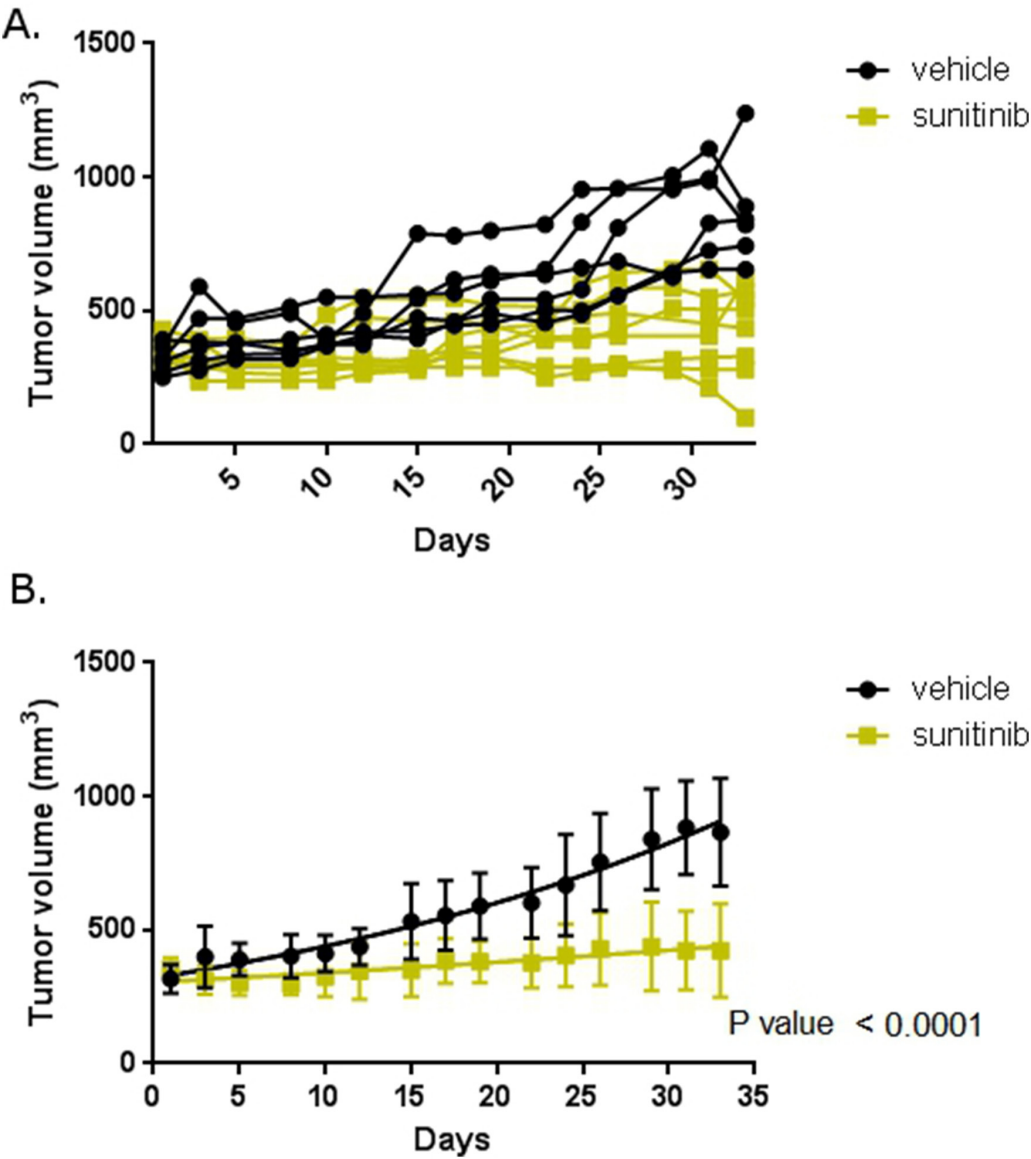
Mouse model reflects clinical experience Mice with xenograft RCC tumors were treated with sunitinib or vehicle. **(A)** Tumor size in individual mice. Mice were treated with vehicle or sunitinib as labelled and tumor size measured with calipers. **(B)** Mean tumor size in each treatment cohort. Error bars are standard deviation. The trend lines are shown within each group. P-value is for difference in slope over the course of the therapy.

To determine the effect of abemaciclib on RCC *in vivo* we employed a mouse model of RCC. We implanted 786-O cells subcutaneously into the flank of nude mice. Mice were monitored for the development of tumors and tumors were measured with calipers. Mice with established, enlarging tumors were then treated with sunitinib or vehicle and tumor size monitored throughout the duration of treatment. Figure 8 shows that mice treated with sunitinib had smaller tumor sizes than mice treated with vehicle over a five week treatment period. This difference is statistically significant. These data establish our model as recapitulating the typical response to sunitinib seen clinically.

**Figure 9:**
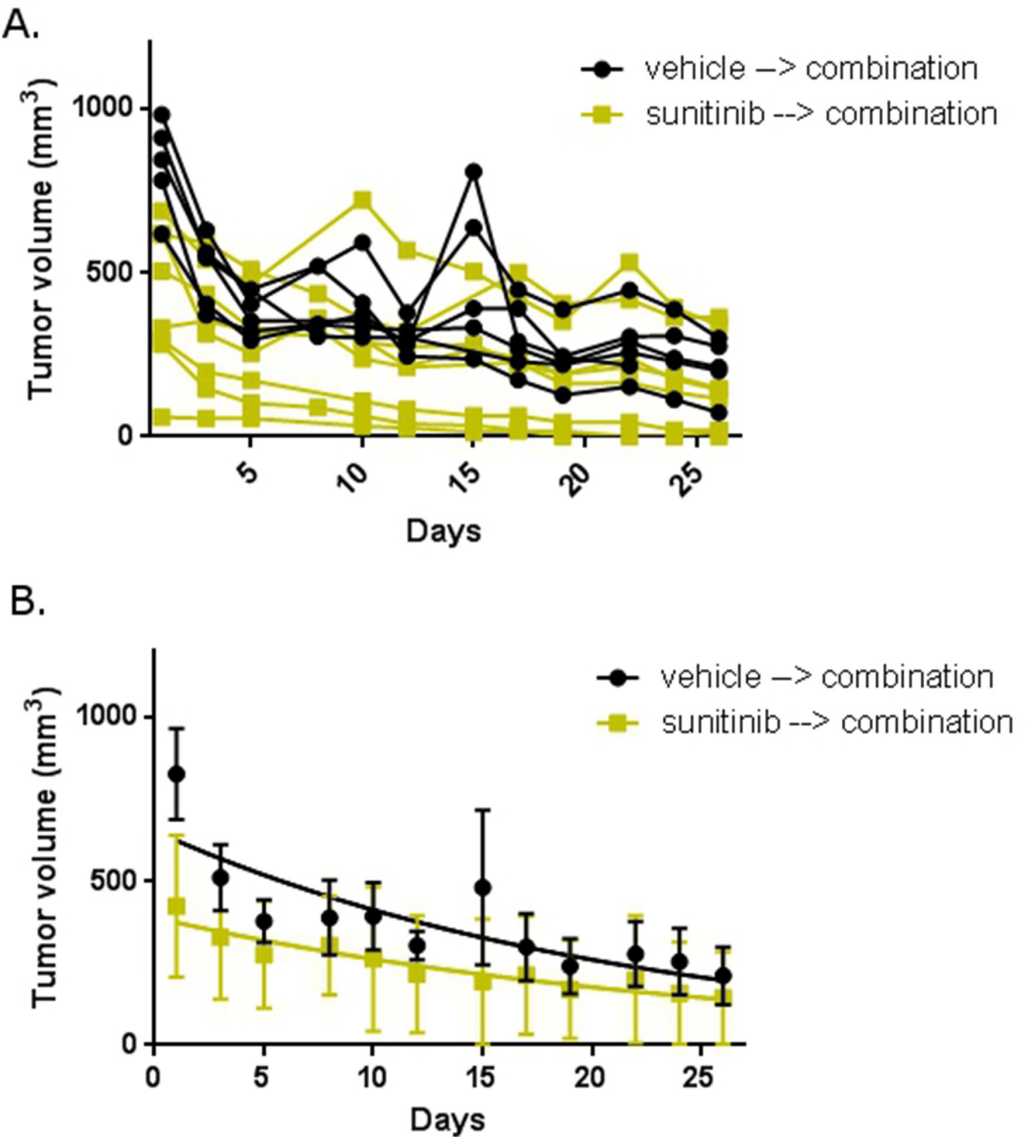
Abemaciclib in combination with sunitinib is active in RCC in mice Mice with xenograft RCC tumors were first treated with sunitinib or vehicle. At the end of treatment, all mice were treated with combination abemaciclib/sunitinib. **(A)** Tumor size in individual mice. **(B)** Mean tumor size in each treatment cohort. Error bars represent standard deviation. The trend lines are shown within each group.

We then explored the *in vivo* effect of combination abemaciclib/sunitinib therapy. After mice completed five weeks of sunitinib or vehicle therapy, all mice were treated with combination abemaciclib/sunitinib for 4 weeks. Tumor sizes were measured throughout the duration of therapy using calipers. Figure 9 shows a rapid reduction in tumor size in mice that had previously received vehicle and in mice that were pre-treated with sunitinib. In both cohorts, tumor sizes continued to decline throughout the duration of therapy. No obvious toxicities were observed. There was no significant weight loss in either cohort, and there were no deaths.

**Figure 10:**
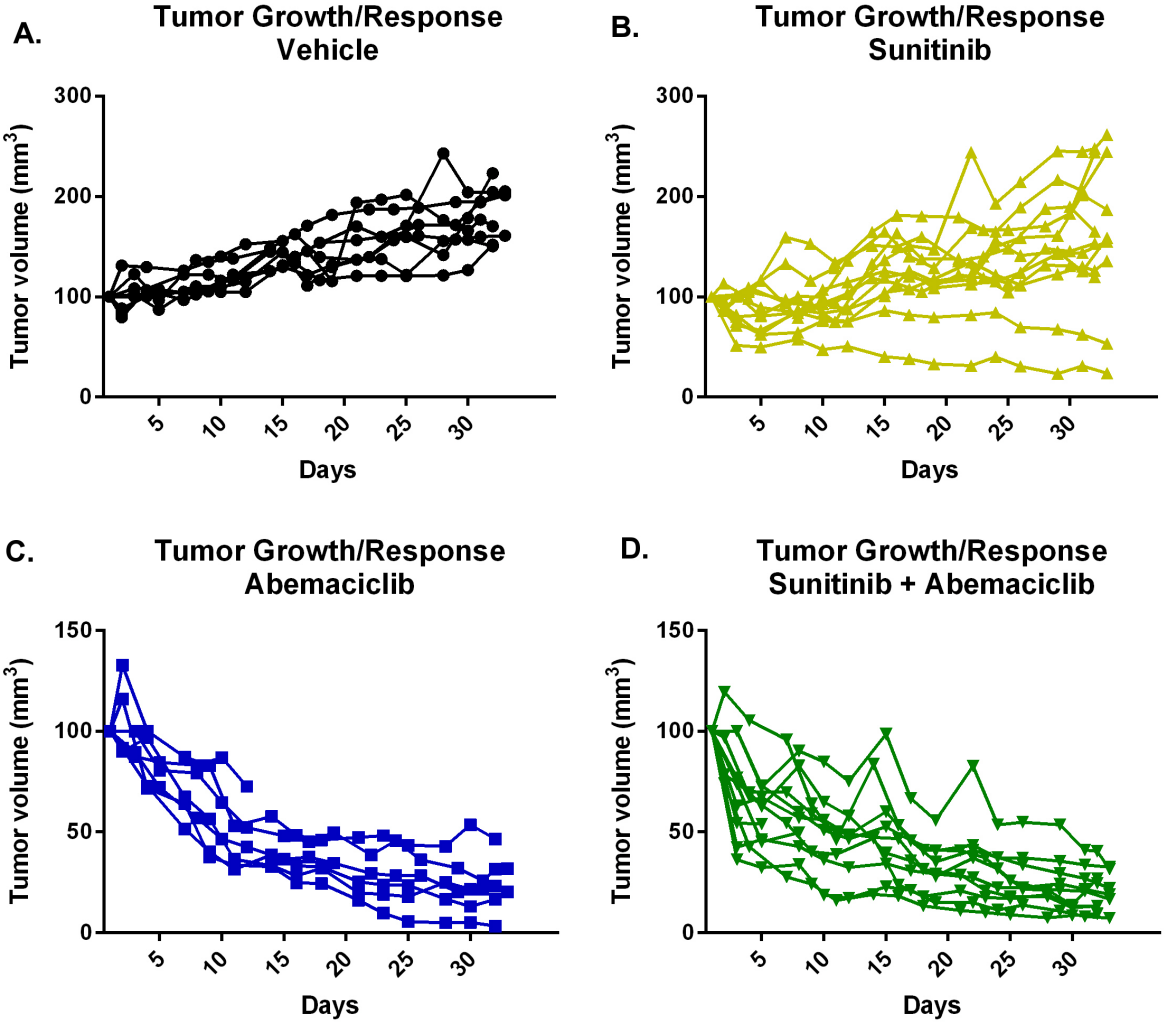
Abemaciclib causes tumor regression as monotherapy and in combination with sunitinib Tumors were established in mice and each cohort treated as labelled. Response to therapy was determined by measurement of tumor size using calipers.

To confirm the *in vivo* activity of combination abemaciclib/sunitinib therapy we performed a second mouse study. Tumors were established in mice as in the prior study. Mice were then treated with vehicle, sunitinib, abemaciclib alone, or combination abemaciclib/sunitinib and tumor response evaluated by measurement with calipers. Figure 10 shows continued tumor growth in control mice and tumor stabilization in most mice undergoing sunitinib therapy. Mice treated with abemaciclib alone show a gradual reduction in tumor size that is maintained throughout the course of therapy. Mice treated with combination abemaciclib/sunitinib show an initial rapid regression of tumor size followed by continued response throughout the course of treatment. These data confirm the results presented in Figure 9 and also suggest single agent activity for abemaciclib in RCC.

**Figure 11:**
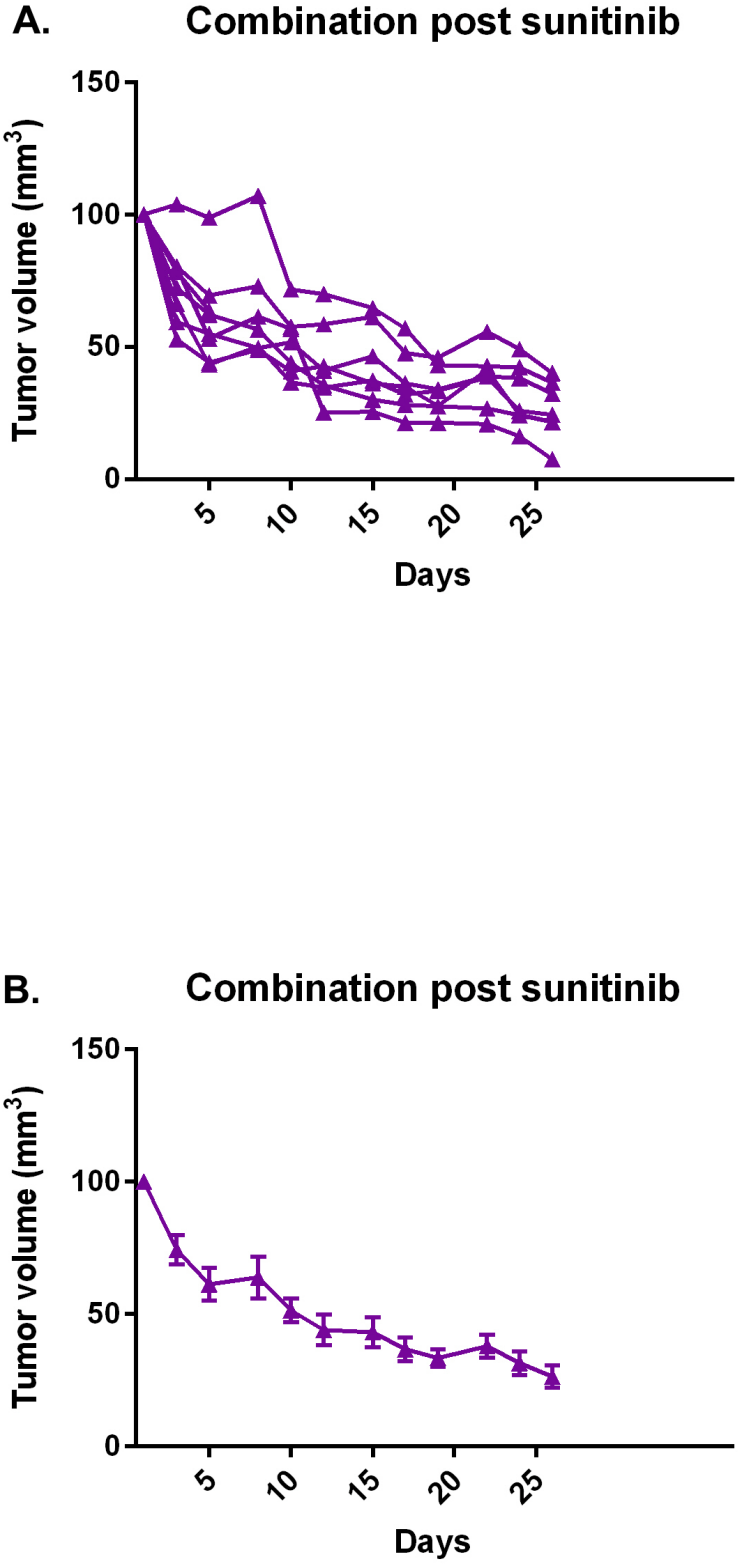
Combination abemaciclib/sunitinib causes tumor regression in mice pre-treated with sunitinib After a course of sunitinib, mice were subsequently treated with combination abemaciclib/sunitinib and tumor response determined by measurement with calipers. **(A)** Individual responses, each line represents an individual mouse. **(B)** Mean response of cohort. Error bars represent standard error of the mean.

As in the prior study, mice treated initially with sunitinib were subsequently treated with combination sunitinib/abemaciclib. Figure 11 demonstrates regression of tumors in the cohort of mice that were pre-treated with sunitinib.

Taken together, our studies demonstrate that combination abemaciclib/sunitinib is an active combination therapy in renal cell carcinoma.

## DISCUSSION

Multiple FDA-approved therapies currently exist for RCC. Current immunotherapies include interleukin-2 [26, 27] and nivolumab [7], while targeted therapies are directed against the VEGF- or mTOR pathways. Here we present data demonstrating that abemaciclib is active against RCC. Abemaciclib is a potent CDK4/6 and PIM1 kinase inhibitor. In prior studies CDK4/6 [19] and PIM1 [15] have both been shown to be potential targets in renal cell carcinoma, making abemaciclib an attractive therapeutic agent. Our data suggest that the targeting of both CDK4/6 and PIM1 is important for the activity of abemaciclib in RCC, as neither SGI-1776 (PIM1 inhibitor) nor palbociclib (CDK4/6 inhibitor) are as potent as abemaciclib in cell culture (see Figure 2).

The improved efficacy of abemaciclib in combination with sunitinib (Figure 3) supports previous work showing that PIM1 inhibition improves the efficacy of sunitinib [15]. The difference in PIM1 staining patterns in RCC and NAT (Figure 1), along with our TMA results showing high levels of PIM1 in RCC but not NAT (Table 1) further validate PIM1 as a target in RCC. Treatment of RCC cell lines with abemaciclib results in increased apoptosis as measured by PARP cleavage (Figure 5) and annexin V staining (Figure 4). PIM1 kinase exerts anti-apoptotic influences within the cell, thus these findings are consistent with the hypothesized PIM1 kinase inhibitory effects of abemaciclib. It is important to note that the apoptotic effects of abemaciclib were increased when used in combination with sunitinib. This observation is consistent with our findings throughout this study that combination abemaciclib/sunitinib is superior to either drug alone in RCC. Furthermore, our data show abemaciclib and sunitinib to have a synergistic effect at low concentrations, further supporting the use of combination therapy.

It is of note that SGI-1776 and abemaciclib inhibit PIM2 kinase, but at much lower potency than PIM1 kinase. SGI-1776 also inhibits PIM3 kinase, while the activity of abemaciclib against PIM3 kinase has not been reported. Future studies will interrogate the PIM family of kinases to determine the degree to which targeting PIM1 is beneficial over targeting PIM2 or PIM3 kinase.

Surprisingly we did not observe abemaciclib-induced effects on cell cycle progression (Supplementary Figure 3). Future studies will focus on further determining the degree to which this mechanism is involved in the effects of abemaciclib on RCC. These studies will include use of additional cell lines and alternative methods for evaluating drug-induced effects on cell cycle progression.

The increase in vacuolization observed in RCC cells treated with abemaciclib (Figure 6) prompted us to evaluate for effects on autophagy. Indeed exposure to abemaciclib increases intracellular LC3b and beclin-1 levels (Figure 7). These changes are time-dependent and at least in the case of beclin-1 appear to have the greatest effect when abemaciclib is used in combination with sunitinib. The increased vacuolization observed with exposure to abemaciclib may represent increased presence of autophagosomes. During autophagy, LC3 is cleaved and converted to the smaller, lower migrating, LC3b, and associates with autophagosomes [28–32]. Consequently, the presence of LC3b has been used as a marker of autophagy [33]. Our data demonstrating an abemaciclib-induced increase in LC3b supports the hypothesis that there is an increase in autophagosomes as a result of abemaciclib exposure. Because beclin-1 is also a marker of autophagy [34–37], abemaciclib-induced increases in beclin-1 levels also suggest an increase in autophagy. Evaluation of additional markers of autophagy will help to elucidate the specific effects of abemaciclib on the autophagic process. Future studies will focus on an evaluation of effects on autophagic flux.

Perhaps the most compelling data we present are the pre-clinical studies of abemaciclib. Our mouse model demonstrates disease stabilization when mice are treated with sunitinib alone (Figure 8). This is the expected outcome and mirrors the effect of sunitinib seen in the clinic [38], suggesting our mouse model is an adequate means of evaluating for initial efficacy of new therapies. Of particular interest is the rapid regression of tumors observed when mice are treated with combination abemaciclib/sunitinib (Figures 9 and 10). Within three days tumor sizes are dramatically decreased and responses continue throughout the course of therapy. It is important to note that responses are observed in treatment-naïve mice, and in mice that were previously treated with single-agent sunitinib.

Our second pre-clinical study confirms the potent activity of combination abemaciclib/sunitinib in our mouse model (Figure 10). Again, responses are seen in treatment-naïve mice and in mice pre-treated with sunitinib. It is notable that responses are also observed in mice treated with abemaciclib alone (Figure 11c), though the responses are not as rapid as with combination therapy.

Taken together, our data show that combination abemaciclib/sunitinib therapy is active in RCC. This is an important finding with multiple implications. Firstly, these data identify new clinical targets in RCC, i.e. CDK4/6 and PIM1 kinase. Secondly, this combination therapy is highly translatable to the clinic. Sunitinib is currently considered standard of care therapy for treatment-naïve metastatic RCC. While abemaciclib is not FDA-approved, it is currently being evaluated in phase III clinical trials in lung and breast cancer populations (NCT02152631, NCT02763566). Earlier phase trials have shown abemaciclib to be both safe and tolerable in humans [22, 39]. Thus we plan a phase Ib study to determine the safety and tolerability of combination sunitinib/abemaciclib in RCC patients, followed by a phase II efficacy study.

The adverse effects of sunitinib are well known and include diarrhea, fatigue, hypertension, hand-foot syndrome, thrombocytopenia, and hypothyroidism [38, 40, 41]. Adverse effects are typically well managed medically with one large study reporting a discontinuation rate for adverse events of 8% [40]. Commonly reported adverse effects of abemaciclib are diarrhea, nausea/vomiting, anorexia, fatigue, leukopenia, anemia, thrombocytopenia, and increased creatinine [22, 39]. In one study in which 173 patients received abemaciclib adverse effects were reversible and there were only two grade 4 adverse events, both for neutropenia [22]. Thus in considering clinical studies using combined abemaciclib/sunitinib therapy we anticipate overlap toxicities to be gastrointestinal (eg. diarrhea) and hematologic (eg cytopenias). Both of these adverse effects are reversible and manageable when these agents are used as monotherapy. A phase Ib study is necessary to determine the degree of adverse effects when these agents are used in combination. In our mouse studies we did not measure blood cell counts, however mice receiving combination therapy were not noted to experience diarrhea or anorexia and there was no significant weight loss compared to mice receiving monotherapy.

In considering clinical studies, our data suggest that combination abemaciclib/sunitinib therapy will be effective in treatment-naïve patients and in patients who are experiencing sunitinib failure. There are numerous agents that are currently FDA approved for use after sunitinib failure and have shown efficacy in that setting, including VEGF-directed therapies, mTOR-directed therapies, and checkpoint inhibitors. In contrast, the current alternatives to sunitinib monotherapy in treatment-naïve disease are few, and include IL-2 (highly toxic, low durable response rate), bevacizumab + interferon (considered non-inferior to sunitinib), pazopanib (considered non-inferior to sunitinib), and perhaps temsirolimus in (poor-risk disease). Use of a first-line regimen that expands the therapeutic targets of treatment outside of the VEGF and mTOR pathways may result in deeper and more durable responses than the currently approved targeted therapies, which may translate into improved disease control and overall survival. Thus we propose evaluating combination abemaciclib/sunitinib therapy in treatment-naïve patients with metastatic clear cell RCC. This, however, need not exclude the very important category of patients experiencing sunitinib failure, and evaluation of combination abemaciclib/sunitinib therapy among those patients is appropriate and desirable.

We are also engaged in mechanistic studies to further elucidate the molecular effects of abemaciclib and combination abemaciclib/sunitinib on cell cycle progression and autophagy. Of particular interest is elucidating the mechanisms that drive improved efficacy with combination therapy over monotherapy.

It is important to note that combination therapy is effective in a VHL-deficient and a VHL-intact RCC cell line. It is a limitation of this study that only two cell lines were used, however these data suggests that combination abemaciclib/sunitinib therapy may have broad efficacy in RCC. The use of additional cell lines will elucidate potential molecular signals that relate to sensitivity of RCC to combination abemaciclib/sunitinib therapy and help to further illuminate important mechanisms of action of the combination therapy.

## MATERIALS AND METHODS

### Immunohistochemistry staining and grading of the TMA

A clear cell renal cell carcinoma TMA unstained slide with 90 cases of tumor and 90 matched NAT cores was obtained from US Biomax, Inc. (HKid-CRC180-01). Immunohistochemistry was performed on a Ventana Discovery XT automated immunostainer using a monoclonal mouse IgG anti-Pim1 antibody (ab75776; Abcam, Cambridge, MA USA; 1:100 dilution). The PIM-1 stained TMA slide was evaluated by two investigators (EW and KM) under multihead microscope. In all 90 cases of tumor and 90 matched NAT cores, the number of moderate to strongly staining nuclei was estimated in a semiquantitative manner on a scale ranging from 0-100%. The staining was graded as: grade 0 (negative, no nuclear staining), 1+ (1 -25% positive nuclei), 2+ (26-50% positive nuclei), 3+ (51-75% positive nuclei), 4+ (76-100% positive nuclei). The individuals assessing the TMA staining were a pathologist and a senior pathology resident, trained in anatomic and clinical pathology and have had extensive experience with renal pathology. They were not informed about the individual cases when grading; however, since they by virtue of professional training and expertise could quickly make the histological diagnoses of cancer vs. NAT, this information carried by the tissue itself could not be hidden.

### Staining and grading of the nephrectomy specimens

Paraffin embedded tissue blocks from 5 nephrectomy specimens for clear cell renal cell carcinoma accessioned between 4/8/15 - 8/12/15 were retrieved from the surgical pathology files of Penn State Hershey Medical Center. No patient had received preoperative chemotherapy or radiation prior to surgical excision. Sections (5 μm thick) were prepared and stained with hematoxylin and eosin and examined under light microscopy. Areas of conventional clear cell renal cell carcinoma were identified according to accepted criteria in all 5 cases. Fuhrman grade for the tissue blocks selected ranged from 2-3. Sections of normal kidney parenchyma with no significant pathologic alteration were identified in each case. The tissue blocks containing the maximum amount of tumor and tissue blocks with no pathological alteration were chosen and sections (5 μm thick) were cut from these for immunohistochemically staining for PIM-1. Immunohistochemistry was performed on a Ventana Discovery XT automated immunostainer using a monoclonal mouse IgG anti-Pim1 antibody (ab75776; Abcam, Cambridge, MA USA; 1:100 dilution). Slides were evaluated by two investigators (KM and EW) under a multihead microscope. For each slide, the number of positively staining cells was estimated in a semiquantitative manner on a scale ranging from 0-100%. The staining was classified as membranous, cytoplasmic, or nuclear. Appropriate approval was obtained from the Penn State Hershey Cancer Institute Institutional Review Board before tissue was obtained for use in this project. The individuals assessing the staining were a pathologist and a senior pathology resident, trained in anatomic and clinical pathology and have had extensive experience with renal pathology. They were not informed about the individual cases when grading; however, since they by virtue of professional training and expertise could quickly make the histological diagnoses of cancer vs. NAT, this information carried by the tissue itself could not be hidden.

### Methylthiazolyldiphenyl-tetrazolium bromide (MTT assays)

786-O (ATCC) and CAKI-1 (ATCC) cells were plated at 5000 cells per well in triplicate in a 96-well tissue culture plate. Cells were left to settle and adhere to the plate overnight in a 37°C/5% CO2 incubator. The next day the desired concentrations of the inhibitors (SGI-1776 = 0, 0.1, 0.5, 1.0, 5.0, 10, 25, and 50 μM; abemaciclib = 0, 0.05, 0.1, 0.5, 1.0, 5.0, 15 μM and 50 μM; palbociclib = 0, 0.1, 0.5, 1.0, 5.0, 10, 15, and 25μM) were calculated and prepared to be added to enough media to attain 200μL per well, of each concentration. SGI-1776 and palbociclib was purchased from selleckchem.com. Abemaciclib was purchased from selleckchem.com or medchemexpress.com. After incubating cells with drug for 21 hours, 20 μL MTT solution was added to each well. MTT solution (5 mg powder in 1 mL sterile DPBS, Sigma Cat# M5655 or suitable manufacturer alternative) was prepared fresh prior to adding to the cells. The cells were incubated with drug for a total of 24 hours, at which time the media was discarded by gently flicking the media in the sink, or suitable container, or paper towels. Then 50 μL DMSO was added to each well by mixing up and down with the pipette (multi-channel) or orbital shaker to dissolve the Formazan crystals. The absorbance at 570 nM of each well was read immediately in a plate reader. The data were analyzed and graphs created using Graphpad Prism 6.05 for Windows, GraphPad Software, La Jolla California USA, www.graphpad.com.

### Luminescent cell viability assays

786-O cells were plated at 5,000 cells per well in a sterile, white opaque-walled/clear bottom, 96-well tissue culture plates (Greiner bio-one, ref# 655098). Cells were left to settle and adhere to the plate overnight in a 37°C/5% CO2 incubator. The next day the desired concentrations of the inhibitors (DMSO = 0.5%; Sunitinib = 5.0 μM; SGI-1776 = 5.0 μM; abemaciclib = 5.0 μM) were added to each plate. Each plate was incubated for 24, 48, or 72 hours as indicated. SGI-1776 and abemaciclib doses were determined based on IC_50_ values of previous dose-response MTT assays and are close to determined IC_50_ values in 786-O cells. Sunitinib showed little activity at 5 μM in 786-O cells in similar dose-response MTT assays (data not shown). For sunitinib we used 5 μM to avoid contamination from activity from monotherapy when assessing for activity when used in combination with SGI-1776 and abemaciclib.

Promega CellTiter-Glo^®^ (CTG) Luminescent Cell Viability Assay was performed on each plate according to manufacturer instructions. Briefly, the CTG Reagent was prepared per manufacturer protocol. Media was discarded from each plate by gently flicking the media in the sink, or suitable container, or paper towels. 100μL of CTG reagent was added to each well and mixed for 2 minutes on an orbital shaker. Plates were then incubated at room temperature for 10 minutes and luminescence subsequently captured using a luminometer.

### Combination index (synergy) analysis

786-O cells were plated in 96-well plates at 5,000 cells per well and exposed to abemaciclib (3.75 μM, 5.625 μM, and 7.5 μM) and sunitinib (3.125 μM, 6.25 μM, 9.375 μM) in combination at the listed concentrations. Cellular viability after 24 hours was determined by MTT assay. The combination index (CI) of each combination was determined using the software Compusyn (verson 1.0). CI values <1 indicate synergism, =1 indicate an additive effect, and >1 indicate antagonism [25].

### Flow cytometry

786-O cells were seeded in p100 petri dishes and allowed to attach overnight. The next day new media containing the desired treatment dose was added to each plate as labelled (0.05% DMSO, 5 μM sunitinib, 5 μM abemaciclib, 5 μM sunitinib + 5 μM abemaciclib). Cells were incubated with drug for 24 hours at 37°C and 5% CO_2_. Annexin V was then evaluated using a phycoerythrin (PE) Annexin V Apoptosis Detection Kit (BD Pharmingen^™^ cat# 559763). The manufacturer protocol was followed. Briefly, cells were detached from the plate using 10mM EDTA in PBS. All subsequent procedures were performed on ice. Cells were washed thoroughly with PBS and resuspended in 1X binding buffer at a concentration of 1 × 10^6^ cells/mL. 100 μL of the solution was transferred to a 5 mL culture tube to which 5 μL of PE Annexin V and 5 μL of 7-AAD was added. Cells were gently vortexed and incubated at room temperature in the dark for 15 minutes. Then 400 mL of 1X binding buffer was added to each tube. Cells were then analyzed by flow cytometry. Flow cytometric data were collected using a BD FACSCalibur (BD Biosciences, San Jose, CA) instrument in the Penn State College of Medicine Flow Cytometry Core Facility.

### Immunoblot assays

The Bolt^™^ electrophoresis system (Life Technologies), which consist of the Bolt^™^ mini gel tank, Bolt^™^ mini precast gels and Bolt^™^ reagents, was used to separate the proteins in the sample lysate. The samples were loaded into the wells of a mini gel and samples run in 1X MES running buffer. After the run was completed, proteins were transferred from the mini gel to a 0.2 mm nitrocellulose membrane using the Bolt^™^ Mini Blot Module (Life Technologies).

When protein transfer was complete the membrane was processed by a standard lab protocol for immunoblot detection of proteins on nitrocellulose membranes. Briefly the membrane was blocked with 5% Milk/1X TBS-0.1% Tween for at least 30 minutes. The immunoblotting chamber was prepared by placing Parafilm M^®^ on the bottom of a plastic western blot box. Wet filter paper was placed on the inside top of the box to help maintain a humid environment during the immunoblotting. Membranes were probed with the following primary antibodies as indicated: PARP (Life Technologies; cat #44698G), GAPDH (Life Technologies; cat #437000), Beclin-1 (Cell Signaling Technologies; cat #3495), and LC3A/B (Cell Signaling Technologies; cat #12741). The membrane was placed on the Parafilm M^®^ in the western box, primary antibody carefully added to cover the membrane, and the membrane placed at 4°C overnight.

The next day the primary antibody solution was removed and discarded, and the membrane washed 3 times for 10 minutes each with 1X TBS-0.1%Tween. Secondary antibody dilutions were prepared in 5% Milk/1X TBS-0.1% Tween (or 5% BSA/1X TBS-0.1% Tween) and the membranes probed with the appropriate secondary antibody: goat anti-rabbit IgG-HRP (Santa Cruz; cat #sc-2004), or goat anti-mouse IgG-HRP (Santa Cruz; sc-2005). Secondary antibody was applied and the membrane incubated at room temperature while rocking for 1 hour. The membrane was then washed 3 times for 10 minutes each with 1X TBS-0.1%Tween.

HyGLO^™^ Chemiluminescent HRP Kit (Denville Scientific Inc.) was used to detect antibody labeled proteins on the membrane. An equal amount of Reagent A was mixed with Reagent B and the solution applied to the membrane for 1 minute. The membrane was then removed from the chemilumiescent solution, placed in an autoradiography cassette and exposed to HyBlot ES^™^ Autoradiography Film (Denville Scientific Inc.). The film was then developed using an atutomated developer.

### Mouse xenograft studies

All animal protocols were approved by the Penn State Milton S. Hershey Medical Center and College of Medicine Institutional Animal Care and Use Committee (IACUC). Human clear cell renal cell carcinoma cells, 786-O (ATCC; cat# CRL-1932), were cultured in RPMI supplemented with 10% fetal bovine serum. We implanted 4×10^6^ cells in 100 μl of a 50/50 mixture of cells in sterile Dulbecco's PBS to Geltrex^™^ LDEV-Free Reduced Growth Factor Basement Membrane Matrix (Life Technologies; cat# A1413202). BALB/c nude mice (Charles River Lab; CAnN.Cg-Foxn1nu/Crl) were anesthetized with isofluorane (5% for induction and 1% for maintenance) for the subcutaneous implantation of 786-O cells into the right flank.

Tumors were allowed to develop and measurements on palpable tumors were performed with a digital caliper. Tumor volume was determined by the ellipsoidal formula, tumor volume = ½ (length x width^2^) [42]. Drug dosing was initiated when tumors reached an easily measurable size, typically 300 – 500 mm^3^. Mice were assigned to each treatment group based on size of tumor at the initiation of therapy. To minimize any effect initial tumor size may have on efficacy we attempted to have equal initial tumor sizes across all treatment groups. Tumors were measured every Monday, Wednesday, and Friday throughout the treatment period. Mice were weighed on the same schedule as tumor measurements.

Mice were treated with vehicle (500 mM Sodium Citrate Buffer, pH 7.0; Boston BioProducts, cat# BB-2036) or sunitinib 40 mg/kg (Medchemexpress; cat# HY-10255). All doses were delivered by oral gavage in a total volume of 150 μL. Drugs were administered daily Monday through Friday, with no dosing on the weekend. The mice were treated for 5 weeks. After 5 weeks of treatment, mice in the vehicle cohort and mice in the sunitinib cohort were treated with the combination of sunitinib 40 mg/kg and abemaciclib 100 mg/kg (HY-16297, Medchemexpress) for an additional 4 weeks. All doses were delivered by oral gavage in a total volume of 150 μL. Drugs were administered daily Monday through Friday, with no dosing on the weekend. After completing therapy mice were euthanized according to IACUC approved protocol. The tumors were surgically removed, fixed in 10% Neutral Buffered Formalin (BDH), and embedded in paraffin. Sunitinib [15, 43] and abemaciclib [21] doses used are consistent with previously published pre-clinical mouse models.

Tumor sizes were recorded in Microsoft Excel version 14 (Redmond, WA, USA) during the course of therapy.

Procedures for the second pre-clinical study were similar to those described above. Mice treated with abemaciclib received 100 mg/kg (HY-16297, Medchemexpress). In the second mouse study mice received sunitinib according to the schedule described above, however abemaciclib was administered daily, including on weekends.

### Statistical methods

Descriptive statistics (such as mean and standard deviation) were used to summarize the numerical measures in this paper. Most of the summary statistics were displayed using graphical methods. A two-way ANOVA model was used to analyze the cell-line data in Figure 3. The time and treatment group interaction effect was examined and found significant. Then within each time point one-way ANOVA model with multiple comparisons were set up to check the pair-wise difference between groups. Multiple comparisons were adjusted using Tukey's method.

For the mouse studies, a linear mixed-effect model was set up to compare the growth curve of tumor volume against time between treatment groups. Statistical analyses were performed using SAS version 9.4 (SAS Institute, Cary, NC, USA) and GraphPad Prism version 6 (GraphPad Software, La Jolla, CA, USA). All tests were two-sided and the statistical significance level used was 0.05.

## SUPPLEMENTARY MATERIALS FIGURES AND TABLES



## Abbreviations

CDK4: cyclin-dependent protein kinase 4
CDK6: cyclin-dependent protein kinase 6
CI: combination index
DMSO: dimethyl sulfoxide
FDA: United States Food and Drug Administration
IACUC: Penn State Milton S. Hershey Medical Center and College of Medicine Institutional Animal Care and Use Committee
IL-2: interleukin-2
LC3: light chain 3
mRCC: metastatic renal cell carcinoma
mTOR: mammalian target of rapamycin
NAT: normal adjacent tissue
PARP: poly ADP-ribose polymerase
PIM1: proviral integration side of Moloney murine leukemia virus 1
RCC: renal cell carcinoma
VEGF: vascular endothelial growth factor
VHL: von Hippel-Lundau.
