# Supplementary material for: The addition of abemaciclib to sunitinib induces regression of renal cell carcinoma xenograft tumors
Source: Oncotarget. 2017 Jul 27;8(56):95116–34. doi: 10.18632/oncotarget.19618 (PMC5707010; doi:10.18632/oncotarget.19618)
Supplement: Supplementary file 1 [file oncotarget-08-95116-s001.pdf]

The addition of abemaciclib to sunitinib induces regression of renal cell carcinoma xenograft tumors

SUPPLEMENTARY MATERIALS

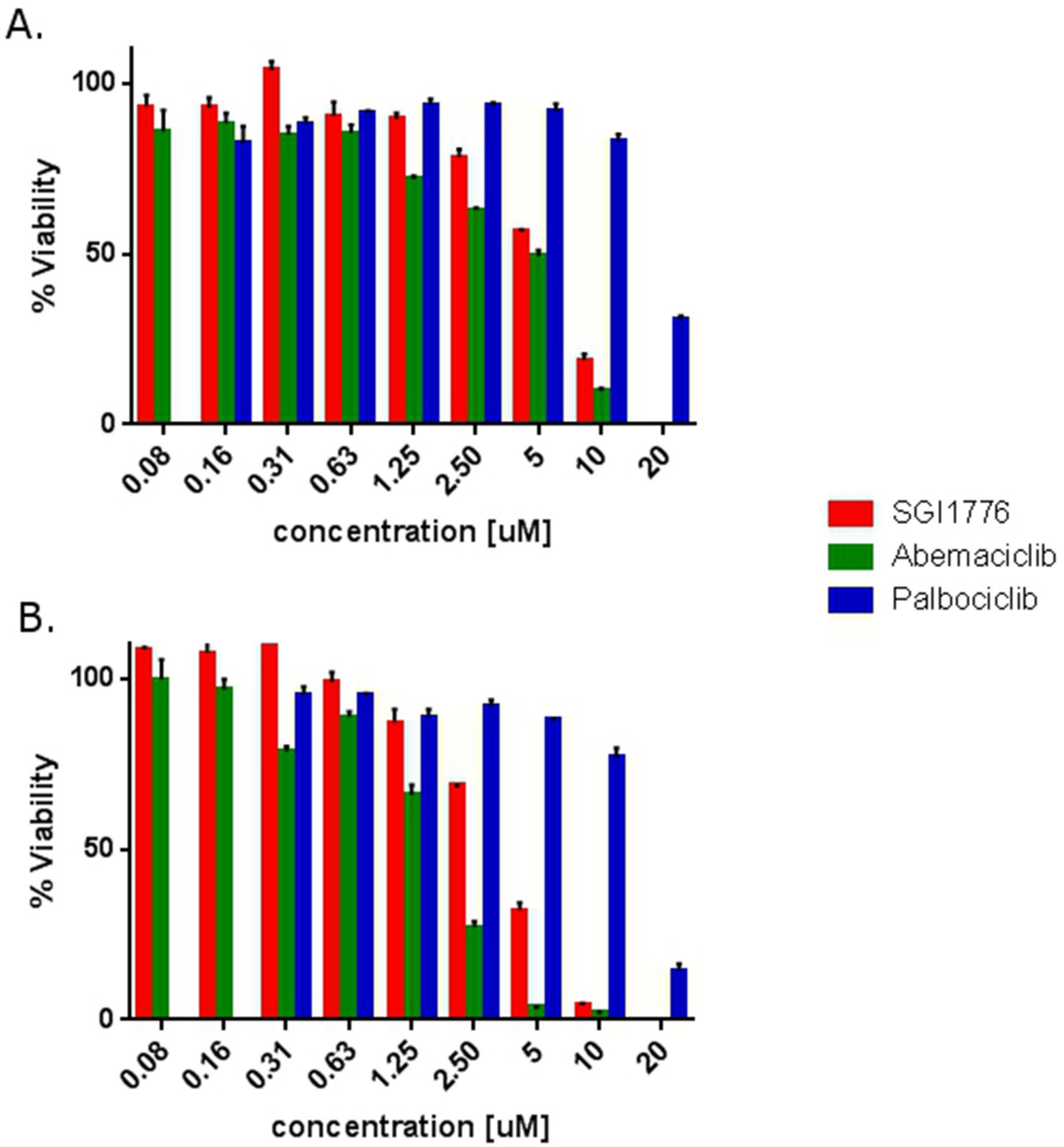

Supplementary Figure 1: Concentration dependent effect of abemaciclib, SGI-1776, and palbociclib in combination with sunitinib in 786-O cells at 24 hours **(A)** and 48 hours **(B)**.

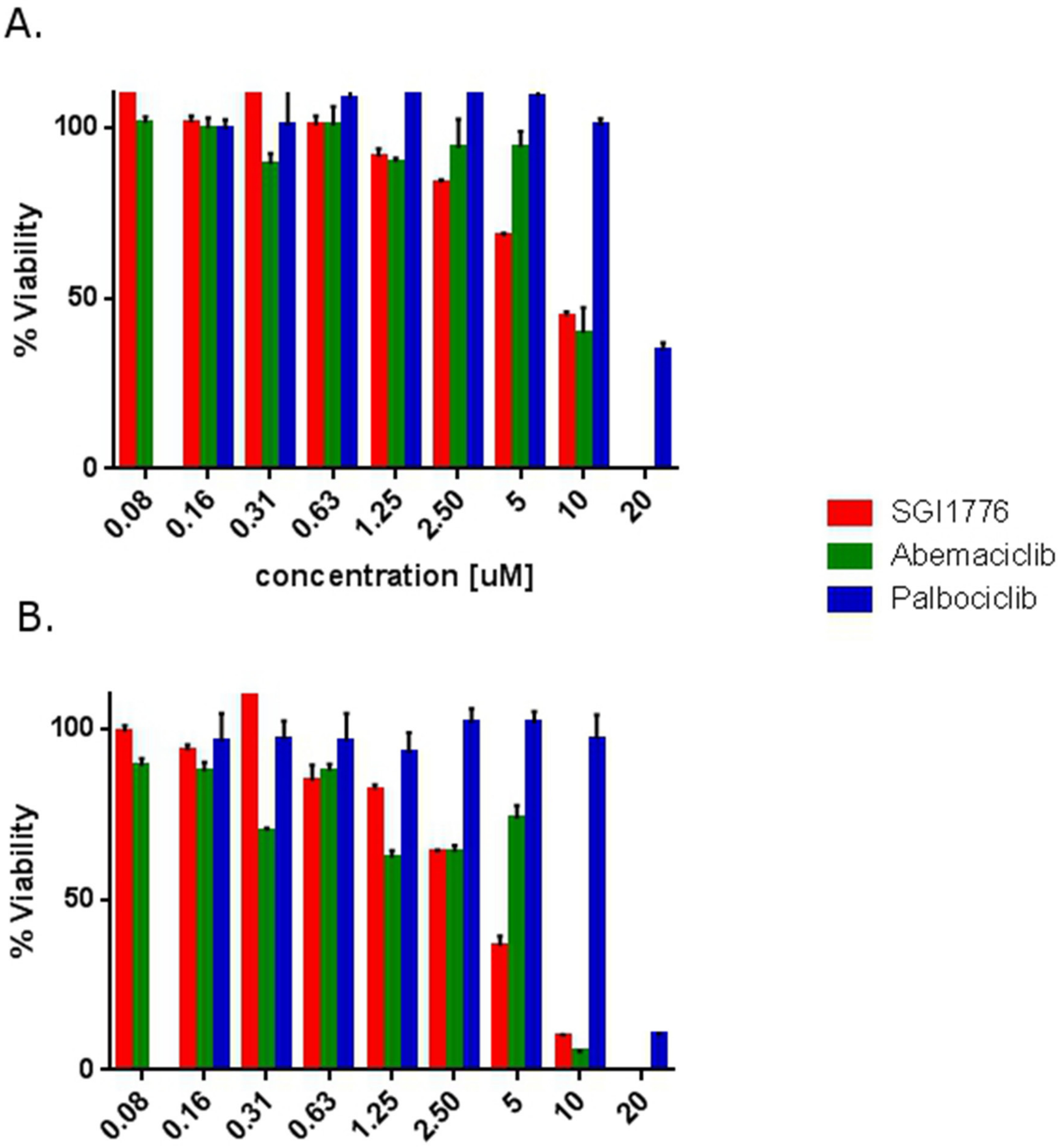

Supplementary Figure 2: Concentration dependent effect of abemaciclib, SGI-1776, and palbociclib in combination with sunitinib in Caki-1 cells at 24 hours **(A)** and 48 hours **(B)**.

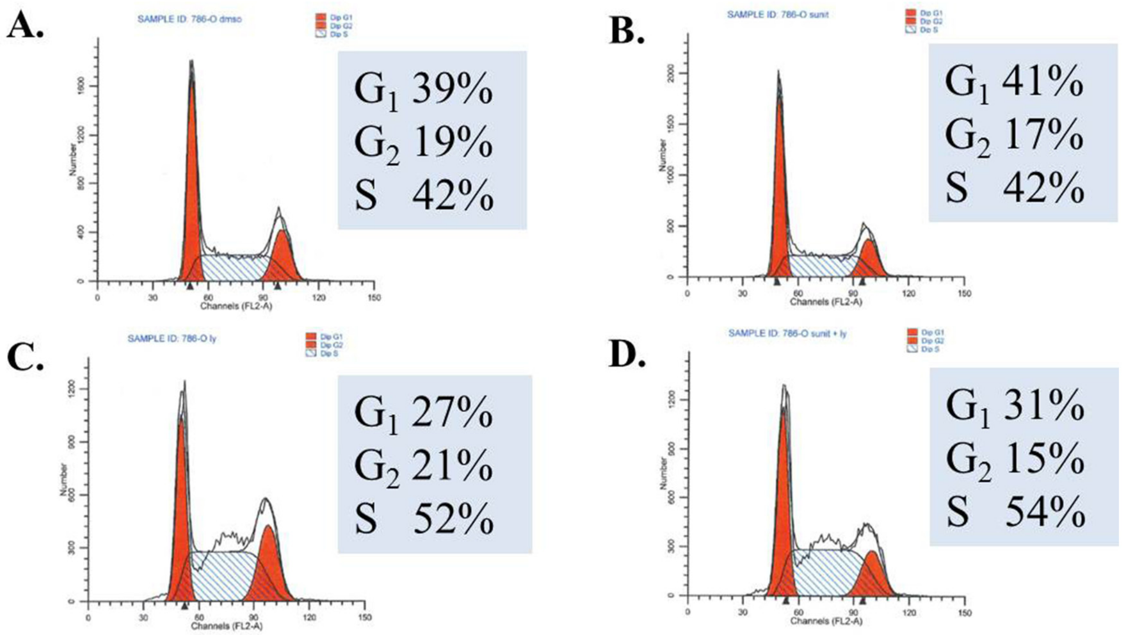

Supplementary Figure 3: Abemaciclib causes an increased population of 786-O cells in S-phase. 786-O cells were treated with DMSO **(A)**, sunitinib **(B)**, abemaciclib **(C)** or the combination of abemaciclib and sunitinib **(D)**. Cell cycle sub-populations were determined by flow cytometry.

Supplementary Table 1: Pairwise comparisons of statistical significance (comparisons are for each figure as labeled)

**Figure 3A**		**2**	**3**	**4**	**5**	**6**
24 hr	1	<.0001	<.0001	<.0001	<.0001	<.0001
	2		0.3343	<.0001	<.0001	<.0001
	3			<.0001	<.0001	<.0001
	4				<.0001	<.0001
	5					<.0001
48 hr	1	<.0001	<.0001	<.0001	<.0001	<.0001
	2		0.4488	0.0098	<.0001	<.0001
	3			0.0005	<.0001	<.0001
	4				<.0001	<.0001
	5					<.0001
72 hr	1	<.0001	<.0001	<.0001	<.0001	<.0001
	2		0.0462	0.6199	<.0001	<.0001
	3			0.0033	<.0001	<.0001
	4				<.0001	<.0001
	5					0.0936
**Figure 3B**		**2**	**3**	**4**	**5**	**6**
24 hr	1	<.0001	<.0001	<.0001	<.0001	<.0001
	2		0.9997	<.0001	<.0001	<.0001
	3			<.0001	<.0001	<.0001
	4				0.0006	0.0005
	5					0.9999
48 hr	1	<.0001	<.0001	<.0001	<.0001	<.0001
	2		0.0017	0.7703	0.0009	0.0005
	3			0.0002	<.0001	<.0001
	4				0.0074	0.0035
	5					0.9970
72 hr	1	<.0001	<.0001	<.0001	<.0001	<.0001
	2		0.4233	0.9997	0.8213	0.7852
	3			0.2393	0.0647	0.0581
	4				0.8898	0.8565
	5					1.0000

Note: groups 1 – 6 are: 1) DSMO, 2) sunitinib, 3) SGI1776, 4) abemaciclib, 5) sunitinib+SGI-1776, and 6) sunitinib+abemaciclib. Values shown are the pair-wise group comparison p-values between groups within each time point. Tukey's methods were used to adjust for multiple comparisons.

Supplementary Table 2: Synergistic activity of abemaciclib and sunitinib in RCC

		sunitinib (uM)		
		3.125	6.25	9.375
abemaciclib (uM)	3.75	0.657	0.878	1.121
	5.625	0.537	0.702	1.018
	7.5	0.825	0.795	1.06

The combination index is shown for each drug combination.
